# Endoscopic treatment of biliary strictures after liver transplantation: balloon dilatation with stenting improves long-term outcomes

**DOI:** 10.1007/s13304-026-02534-9

**Published:** 2026-02-06

**Authors:** Xiaofang Zhang, Guangkai Zhao, Zhongxin Huang, Zeliang Xu, Di Jiang, Danqing Liu, Leida Zhang, Chengcheng Zhang, Xi Wang

**Affiliations:** 1 Department of Hepatobiliary Surgery, Southwest Hospital, Third Military Medical University (Army Medical University), No.30, Gaotanyan Road, Chongqing, 400038 China; 2 Department of Organ Transplantation Surgery, Liaocheng People’s Hospital/Affiliated Liaocheng Hospital, Shandong First Medical University, Shandong, China; 3 Department of Radiology, Women and Children’s Hospital of Chongqing Medical University, Chongqing, China

**Keywords:** Biliary strictures, Endoscopic retrograde cholangiopancreatography, Liver transplantation, Overall survival, Balloon dilatation

## Abstract

Endoscopic treatment is the standard treatment for biliary strictures after liver transplantation (LT), but the specific strategies and different success patterns have not been clearly defined in terms of their impact on survival. The aim of this study was to investigate the predictors of successful endoscopic treatment and the impact of different patterns of success on patient survival outcomes. A total of 93 patients with accurately diagnosed biliary strictures who subsequently underwent endoscopic retrograde cholangiopancreatography (ERCP) were retrospectively enrolled. The primary endpoint was standard of care success and final treatment success, while secondary endpoint was overall survival. Non-anastomotic strictures (NAS) and anastomotic strictures (AS) were diagnosed in 57 and 36 patients, respectively, based on imaging before the last ERCP procedure. Definitive treatment success was achieved significantly more often in patients with AS (*P* = 0.018), while there was no significant difference in standard treatment success between AS and NAS (*P* = 0.900). Both treatment success patterns showed a better overall survival benefit in the overall and NAS groups (*P* < 0.05), and in the AS group, the survival curve showed a long-term benefit for both treatment success patterns, despite no statistical difference. In addition, balloon dilatation with stenting increased final treatment success in the overall groups. ERCP is a safe and effective therapeutic option for patients with NAS and AS, with both successful treatment patterns improving long-term survival. Balloon dilatation with stenting can increase the treatment success of ERCP in both types of biliary strictures.

**Graphical abstract:**

**Figure:**
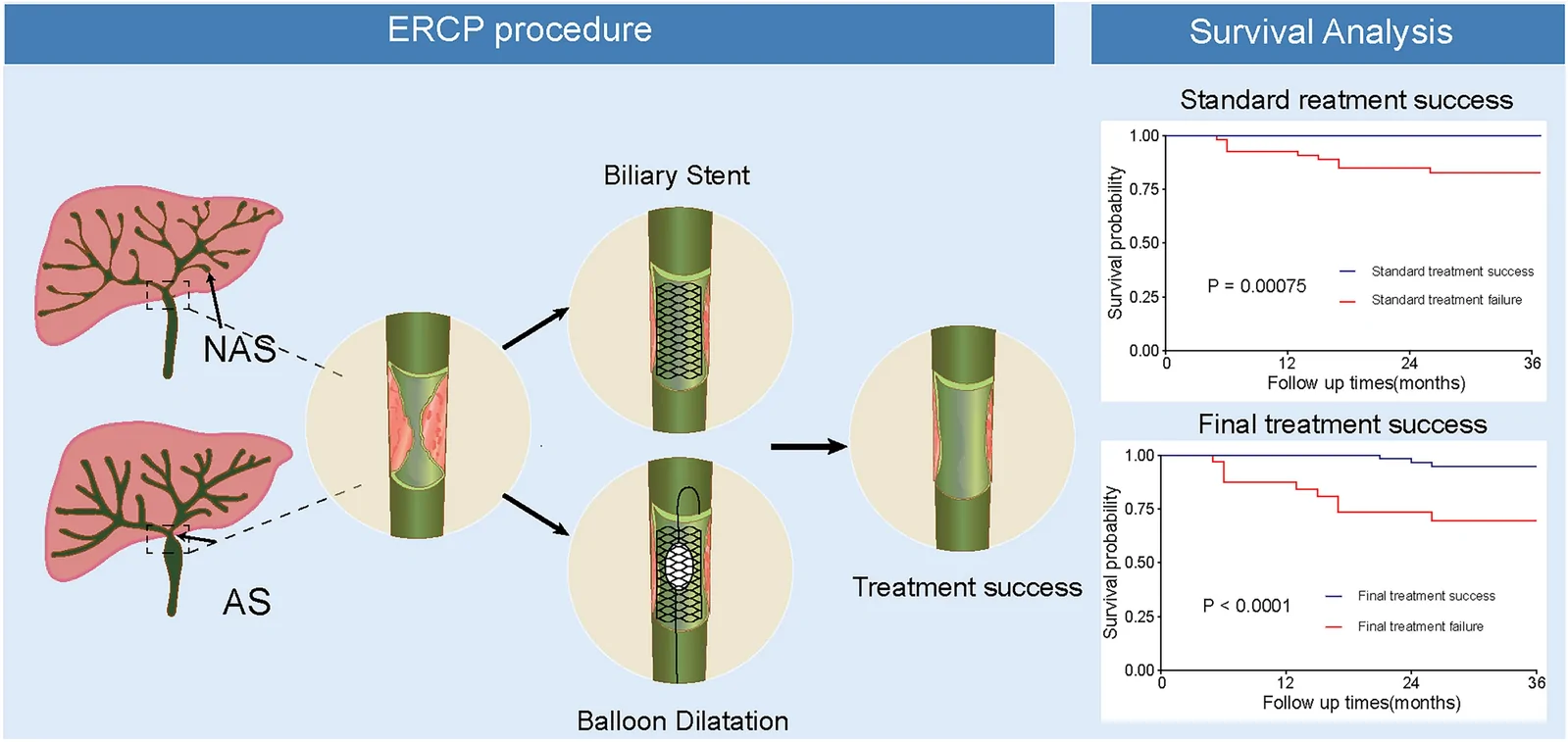

**Supplementary Information:**

The online version contains supplementary material available at https://doi.org/10.1007/s13304-026-02534-9.

## Introduction

Biliary complications, particularly biliary strictures, are the most common category of complications following liver transplantation (LT) and are a major determinant of graft function and patient survival [1, 2]. Approximately 80% of strictures typically manifest within the first year after LT and are classified into non-anastomotic strictures (NAS) and anastomotic strictures (AS) according to their anatomical location [3]. In general, AS are more common than NAS and therapeutic management is relatively straightforward. However, as the disease progresses, some patients with AS may progress to NAS [4, 5]. This transition may be due to various factors such as persistent inflammation, fibrosis or changes in biliary anatomy secondary to the initial transplantation procedure or subsequent interventions [6–8]. Therefore, early diagnosis and intervention are essential to prevent further deterioration of graft function and optimize patient outcomes.

Endoscopic retrograde cholangiopancreatography (ERCP) is widely regarded as the primary therapeutic intervention for the management of biliary strictures [9, 10]. In instances where ERCP is unsuccessful or anatomical alterations necessitate an alternative approach, percutaneous transhepatic biliary drainage (PTCD) is employed. Surgical reintervention is reserved for cases where interventional therapeutic measures have failed [11]. It is evident that the disparate positioning of strictures necessitates the implementation of disparate ERCP treatment protocols for AS and NAS. The standardized therapeutic regimen typically lasts at least 12 months for AS and NAS often requires long-term treatment, with a standardized treatment duration of 2 years [8, 12].

In the case of AS, the standard endoscopic management strategy involves biliary sphincterotomy in conjunction with balloon dilation and the insertion of multiple plastic stents (MPS) [13, 14]. The MPS frequently necessitates periodic replacement at intervals of approximately 3 months to mitigate the risk of stent occlusion and the development of cholangitis. This process continues until the stricture resolves completely. Despite a stricture resolution rate of up to 95%, this methodology imposes a significant economic and physiological burden on patients due to the necessity of multiple ERCPs [6]. In this situation, fully covered self-expanding metal stent (FCSEMS) has emerged as an alternative. A lot of evidence from multiple studies indicates that FCSEMS exhibits a comparable efficacy in relieving stricture to that of MPS [15–17]. Furthermore, it has been demonstrated to reduce the number of ERCPs, the incidence of postoperative adverse reactions, and the complexity of the procedure. The most recent guidelines recommend the use of FCSEMS as the preferred stent for patients with biliary strictures following LT [18, 19]. However, the quality of the evidence is not high, leading to controversy. Compared to AS, ERCP treatment for NAS is more complex and challenging, and there is currently no standardized treatment guideline. Similar to AS, MPS has been proven to be successful in the treatment of NAS, but the stricture relief rate is only 35–65% [6], mainly from small study cohorts and short-term follow-up studies. Overall, patients with NAS have a lower success rate, greater difficulty, and longer duration of ERCP treatment.

In conclusion, the decision-making process for ERCP treatment diverges between NAS and AS, owing to their distinct etiologies. The aim of our retrospective research is to elucidate the key factors that predict the achievement of standard ERCP treatment success and ultimate treatment success, as well as to dissect the ramifications of both success patterns on patient survival outcomes.

## Methods

### Study design and study population

This retrospective study included adult LT patients (> 18 years) from Southwest Hospital between January 2015 and January 2023. All donor and recipient data were recorded in the Chinese Liver Transplant Registry (https://cltr.cotr.cn), and organs were obtained only from deceased donors. This study adhered to the tenets of the Declaration of Helsinki and institutional ethics guidelines, and was approved by ethics committees. Informed consent was not required due to the retrospective design. The final study population included only patients diagnosed with NAS and/or AS who underwent ERCP as a subsequent treatment. Patients without a definitive diagnosis of NAS or AS or those with a confirmed diagnosis but who refused ERCP treatment were also excluded from the study. Follow-up was completed by October 2024, with an average duration of 51.2 months.

### Data collection

Transplantation features were collected as follows: Recipient age, recipient sex, recipient BMI, donor type categorized as donation after circulatory death (DCD) or donation after brain death (DBD), liver disease, utilization of Basiliximab, Split-LT, salvage LT (SLT), gastroduodenal artery (GDA) disconnection, biliary stent implantation, cold ischemia time (CIT), strictures onset time post-LT, and major complications (Clavien-Dindo grade not exceeding III). ERCP treatment features included duration of ERCP treatment, ERCP frequency, stents implantation frequency, type of stents, balloon dilatation, adverse events of stents implantation, adverse events of ERCP, and with/without bile leakage or stones. Furthermore, laboratory parameters before LT, initial and last ERCP as well as at the end of the follow-up period were collected, including white blood cell (WBC), hemoglobin (Hb), platelet (PLT), alanine aminotransferase (ALT), aspartate aminotransferase (AST), albumin (ALB), creatinine (Cr), prothrombin time (PT), total bilirubin (TBIL).

### Definitions

All patients received regular follow-up after LT. When patients exhibit clinical manifestations such as elevated cholestatic laboratory parameters, progressive jaundice, and cholangitis, Magnetic Resonance Cholangiopancreatography (MRCP) was performed to confirm the diagnosis. It is noteworthy that all diagnosis of biliary stricture were made on the basis of MRCP prior to the final ERCP procedure, rather than relying solely on the initial diagnostic assessment. NAS was defined as stricture of the affected hepatic bile duct, located at least 1 cm away from the anastomotic site; AS is defined as stricture at or near the site of biliary anastomosis [11].

### ERCP procedure

Patients with clinical symptoms or abnormal laboratory tests suggesting biliary strictures should be recommended for ERCP. All ERCP procedures were performed by experienced endoscopic experts at the Southwest Hospital Endoscopy Centre. Stenting was chosen as the preferred treatment, with balloon dilatation or catheter dilatation only performed if the stent could not pass through the stricture. The type of stent was chosen independently, based on the experience of different endoscopists. Repeat ERCP was routinely performed 3 months after MPS implantation or 6 months after FCSEMS implantation. ERCP was performed immediately in cases of worsening cholestasis or clinical symptoms, or in cases of acute cholangitis. As long as the clinical symptoms disappeared, the laboratory indicators tended to be normal and the cholangiography suggested that the stricture was relieved or completely resolved, the stent removal procedure should be performed and regular follow-up should be performed within 1 year.

### Study outcomes

The primary outcome of this study was the standard treatment success and final treatment success. In short, final treatment success was defined as no intervention within 12 months after the last ERCP treatment, including ERCP, retransplantation and biliary reconstruction, normal laboratory parameters, and no clinical symptoms related to cholangitis or jaundice. Based on this, NAS treatment duration within 2 years and AS treatment duration within 1 year were defined as standard treatment success. The secondary outcome of this study was overall survival of patients and treatment failure. Besides, Patients were also regarded as treatment failure if they relapsed after previous treatment success and required intervention during the next 12 months of follow-up.

### Statistical analysis

Continuous variables were presented as mean ± standard deviation and were compared with Student’s tests. Categorical variables were presented as frequencies and percentages and were compared with the χ^2^ tests or Fisher’s test. Univariate logistic regression analysis was used to select the factors associated with standard treatment success and final treatment success. K-M survival curves were plotted for survival analysis. The log-rank test was used to calculate differences in actuarial survival. A two-tailed P value < 0.05 was considered statistically significant. Data analyses were conducted on R 3.6.3 (R Foundation for Statistical Computing, Vienna, Austria) and SPSS 26.0 (IBM Corp., Armonk, NY, USA).

## Results

### Baseline characteristics

From January 2015 to January 2023, a total of 97 adult LT recipients were ultimately diagnosed with NAS or AS at Southwest Hospital. Four patients were excluded from the analysis: three patients refused ERCP treatment, and one patient was excluded due to lost follow-up data. Therefore, the final research cohort consisted of 93 patients, including 57 with NAS (61.3%) and 36 with AS (38.7%). The median time to stricture onset after LT was 9.8 months for the overall group, 10.0 months for the NAS group, and 9.5 months for the AS group. The median total follow-up period was 61.0 months for the overall group, 52.6 months for the NAS group, and 58.6 months for the AS group (Table 1). Additionally, this study examined laboratory parameters at pre-transplantation, before the initial and final ERCP treatments, and at the end of the follow-up period (Supplement Table S1).

**Table 1:** Patient characteristics and transplantation features

Characteristics	Overall group N = 93	NAS group N = 57	AS group N = 36
Incidence	93/389 (23.9)	57/389 (14.7)	36/389 (9.2)
Age (years)	49.2 ± 10.1	48.9 ± 9.8	49.6 ± 10.7
BMI (kg/ m^2^)	23.5 ± 3.8	23.5 ± 3.8	23.6 ± 3.7
Cold ischemia time (h)	6.5 ± 1.5	6.5 ± 1.6	6.3 ± 1.4
Strictures onset time post-LT (months)	9.8 ± 10.8	10.0 ± 11.7	9.5 ± 9.5
Males	79 (84.9)	48 (84.2)	31 (86.1)
Liver disease			
Viral	42 (45.2)	28 (49.1)	14 (38.9)
Hepatocellular carcinoma	31 (33.3)	14 (24.6)	17 (47.2)
Autoimmune liver diseases	5 (5.4)	3 (5.3)	2 (5.6)
Other	15 (16.1)	12 (21.1)	3 (8.3)
Basiliximab (+)	65 (69.9)	40 (70.7)	25 (69.4)
Split-LT	11 (11.8)	6 (10.5)	5 (13.9)
Salvage LT	11 (11.8)	5 (8.8)	6 (16.7)
Donor type (DBD)	20 (21.5)	9 (15.8)	11 (30.6)
GDA disconnection	34 (26.6)	23 (40.4)	11 (30.6)
Biliary stent implantation during LT	3 (3.2)	2 (3.5)	1 (2.8)
Major complications post LT	13 (14.0)	7 (12.3)	6 (16.7)

Continuous variables are reported as median and range

Categorical variables are provided as counts and percentages

NAS, non-anastomotic strictures; AS, anastomotic strictures; BMI, body mass index; LT, liver transplantation; DBD, donation after brain death; GDA, gastroduodenal artery

### ERCP treatment procedure

**Fig. 1:**
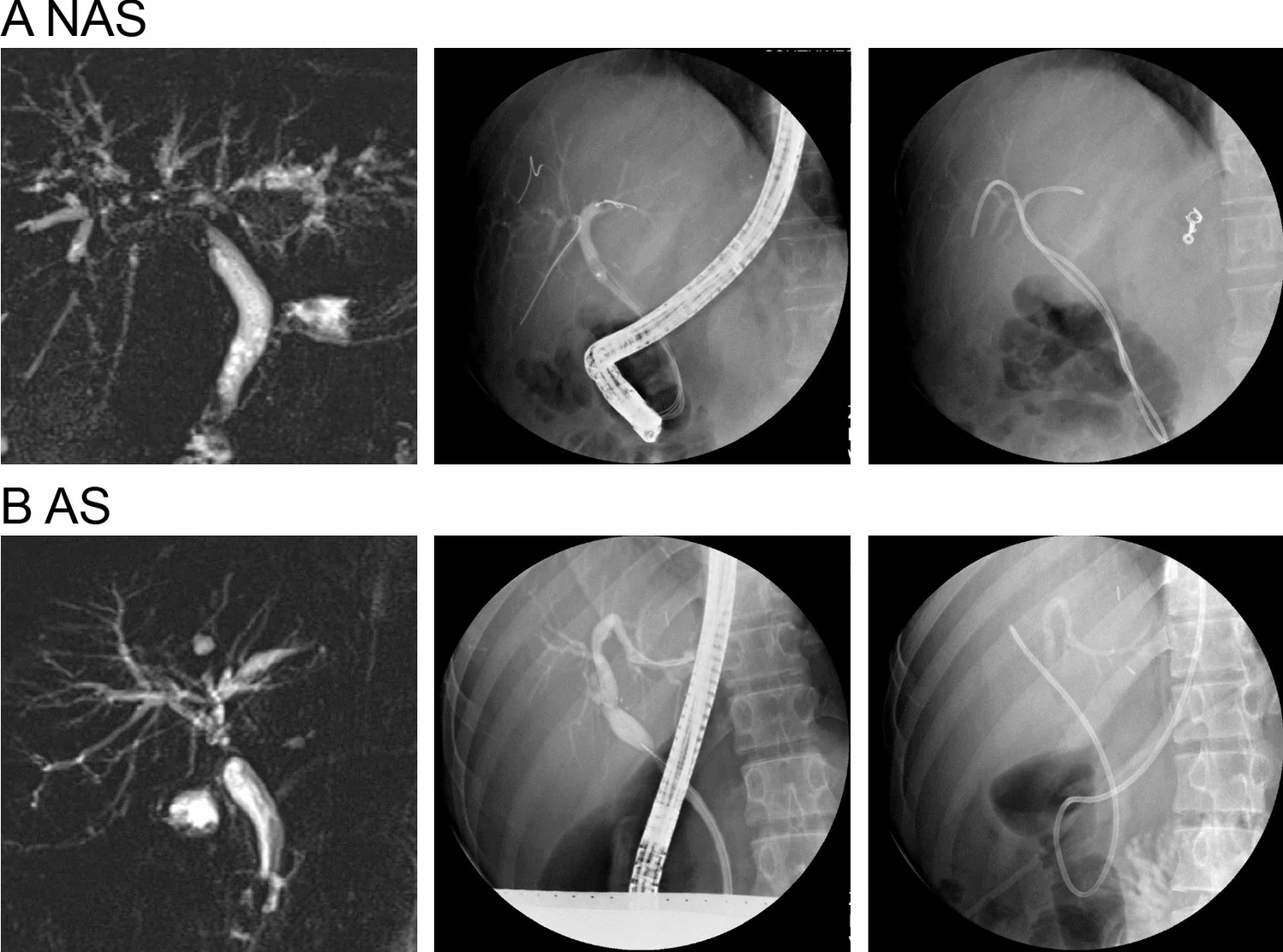
MRCP and ERCP images of NAS group (**A**) and AS group (**B**)

**Fig. 2:**
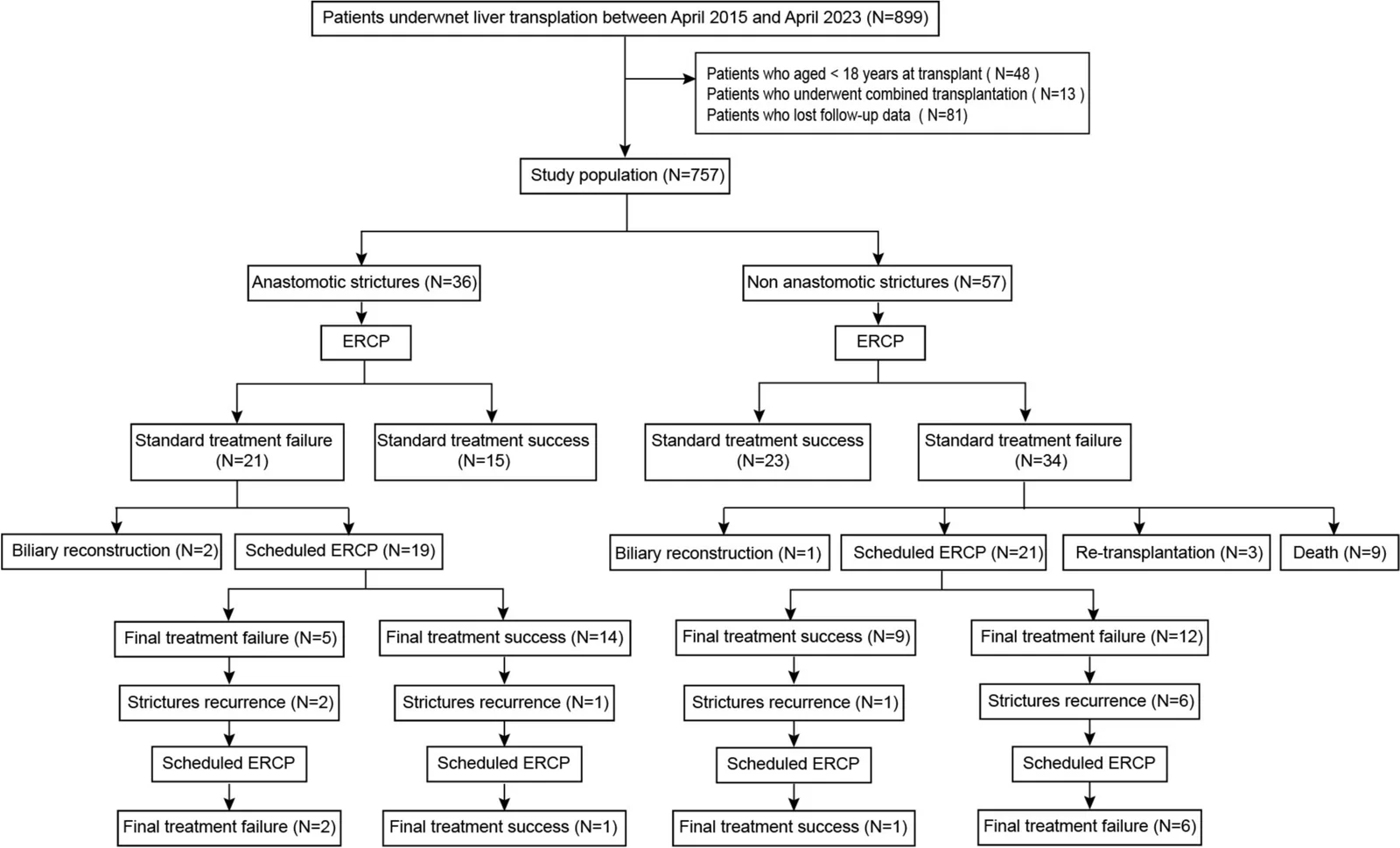
ERCP treatment procedure in patients with AS and NAS

All patients underwent ERCP treatment immediately after a clear diagnosis of NAS or AS (Fig. 1). A total of 48 patients achieved standard treatment success (NAS group: N = 23/57; AS group: N = 15/36). There were no stricture recurrences during follow-up and 45 patients (48.4%) achieved standard treatment failure. In the NAS group, 9 patients died, 3 patients underwent retransplantation, 1 patient underwent biliary reconstruction and the other 21 patients continued to receive ERCP treatment. Of these, 9 patients ultimately achieved treatment success, including 1 patient who achieved final treatment success after recurrence, and 12 patients experienced treatment failure, including 6 patients who experienced treatment failure after recurrence. In the AS group, 2 patients underwent biliary reconstruction and 19 patients continued ERCP treatment. Of these, 14 patients ultimately achieved treatment success, including 1 patient who relapsed and achieved treatment success again, and 5 patients failed treatment, including 2 patients who had treatment failure after relapse (Fig. 2, Table 2).

**Table 2:** P ERCP treatment features and outcomes

Characteristics	Overall group N = 93	NAS group N = 57	AS group N = 36
Duration of ERCP treatment (months)	17.2 ± 16.7	19.2 ± 18.1	14.0 ± 13.8
Duration of Follow-up (months)	51.2 ± 28.9	52.6 ± 29.9	49.1 ± 27.7
No. of ERCP	3.8 ± 2.5	4.5 ± 2.8	2.6 ± 1.3
No. of stents	3.0 ± 2.4	3.8 ± 2.6	1.8 ± 1.1
Type of stents			
MPS	58 (62.4)	48 (84.2)	10 (27.8)
FCSEMS	17 (18.3)	2 (3.5)	15 (41.7)
Both	18 (19.4)	7 (12.3)	11 (30.6)
Balloon dilatation	73 (78.5)	50 (87.7)	23 (63.9)
Adverse events of stents	14 (15.1)	9 (15.8)	5 (13.9)
With bile leakage	3 (3.2)	1 (1.8)	2 (5.6)
With stones	72 (77.4)	48 (84.2)	24 (66.7)
Adverse events of ERCP	25 (26.9)	19 (33.3)	6 (16.7)
Standard treatment success	48 (51.6)	23 (40.4)	15 (41.7)
Treatment success	61 (65.6)	32 (56.1)	29 (80.6)
Treatment failure	32 (34.4)	25 (43.9)	7 (19.4)
Re-transplantation	3 (3.2)	3 (5.3)	0 (0)
Biliary reconstruction	3 (3.2)	1 (1.8)	2 (5.6)
Death	9 (9.7)	9 (15.8)	0 (0)
Recurrence of biliary strictures	10 (10.8)	7 (12.3)	3 (8.3)
Successful treatment of recurrence	2 (2.2)	1 (1.8)	1 (2.8)

Continuous variables are reported as median and range

Categorical variables are provided as counts and percentages

NAS, non-anastomotic strictures; AS, anastomotic strictures; ERCP, endoscopic retrograde cholangiopancreatography; MPS, multiple plastic stents; FCSEMS, fully covered self-expanding metal stent

### Success rates and outcomes

The standard treatment success rate for the overall group was 51.6% (48/93), with 40.4% (23/57) for the NAS group and 41.7% (15/36) for the AS group. For the final outcomes, the treatment success rate was 65.6% (61/93) for the overall group, 56.1% (32/57) for the NAS group and 80.6% (29/36) for the AS group. The frequency of ERCP for the overall group was 3.8 ± 2.5, for the NAS group was 4.5 ± 2.8 and for the AS group was 2.6 ± 1.3. All patients underwent internal stenting, with an overall stenting frequency of 3.0 ± 2.4 in the overall group, 3.8 ± 2.6 in the NAS group and 1.8 ± 1.1 in the AS group. The incidence of adverse events after stent implantation was 15.1% (14/93) in the overall group, 15.8% (9/57) in the NAS group and 13.9% (5/36) in the AS group. In addition, the incidence of bile leakage during ERCP was 3.2% (3/93) in the overall group, 1.8 (1/57) in the NAS group and 5.6 (2/36) in the AS group, while the incidence of stones was 77.4%, 84.2% and 66.7% in the overall group, NAS group and AS group, respectively. A total of 25 patients (26.9%) in the overall group experienced ERCP-related adverse events (NAS group: N = 19; AS group: N = 6), including bacterial cholangitis (N = 15) and post-ERCP pancreatitis (N = 10). All ERCP-related adverse events were controlled with conservative measures and no patients died (Table 2).

The trends in liver function indicators such as ALT, AST, ALB, TBIL, Cr and PT over time were then plotted for patients with and without achieved successful standard treatment and final success treatment at overall group (Supplement Fig. S1), NAS group (Supplement Fig. S2) and AS group (Supplement Fig. S3). This study found that regardless of the function before transplantation and ERCP, the time trend of liver function indicators in the standardized treatment success group and final treatment success group was better than that in the failure group.

### Predictors for standard treatment success and final treatment success

For standard ERCP treatment outcomes, in the overall group, the number of stents implanted was significantly associated with standard treatment failure (*P* < 0.05). Whereas in AS, apart from the number of stents implanted, multiple ERCPs were significantly associated with standard treatment failure (*P* < 0.05). However, for final ERCP treatment outcomes, both MPS and FCSEMS implantation and balloon dilatation significantly increased the likelihood of final treatment success in the overall group and NAS group, and NAS was significantly associated with treatment failure in the overall group (*P* < 0.05). Besides, balloon dilatation also increased the likelihood of final treatment success in the AS group (*P* < 0.05, Table 3).

**Table 3:** Predictors of patients with achievement of standard treatment success and treatment success at overall group, NAS group and AS group

	Overall groups				NAS groups			AS groups		
	OR	95%CI	P value^a^		OR	95%CI	P value^a^	OR	95%CI	P value^a^
Standard treatment success										
Biliary Stricture (NAS)	0.947	0.406–2.211		0.900	-	-	-	-	-	-
No. of ERCP	0.823	0.676–1.003		0.054	0.871	0.705–1.076	0.201	0.451	0.226–0.897	0.023
No. of stents	0.793	0.638–0.987		0.038	0.829	0.656–1.049	0.118	0.272	0.090–0.825	0.021
Type of stents										
MPS	Ref	Ref		Ref	Ref	Ref	Ref	Ref	Ref	Ref
FCSEMS	0.862	0.288–2.577		0.790	-	-	0.999	0.583	0.115–2.952	0.515
Both	0.473	0.149–1.501		0.204	1.050	0.211–5.217	0.952	0.148	0.020–1.081	0.060
Balloon dilatation	1.047	0.382–2.870		0.930	4.714	0.528–42.098	0.165	0.457	0.114–1.831	0.269
Adverse events of stents	0.529	0.153–1.833		0.316	0.700	0.156–3.137	0.641	0.304	0.030–3.036	0.310
With bile leakage	0.716	0.063–8.192		0.788	-	-	1.000	-	-	1.000
With stones	1.161	0.428–3.148		0.770	1.429	0.319–6.402	0.641	1.000	0.245–4.078	1.000
Adverse events of ERCP	1.863	0.738–4.707		0.188	2.137	0.695–6.566	0.185	1.500	0.258–8.711	0.651
Final treatment success										
Biliary Stricture (NAS)	0.309	0.116–0.821		0.018	-	-	-	-	-	-
No. of ERCP	1.124	0.928–1.362		0.231	1.211	0.973–1.507	0.087	2.104	0.875–5.063	0.097
No. of stents	1.055	0.873–1.275		0.577	1.160	0.931–1.445	0.186	2.526	0.661–9.662	0.176
Type of stents										
MPS	Ref.	Ref.		Ref.	Ref.	Ref.	Ref.	Ref.	Ref.	Ref.
FCSEMS	1.818	0.567–5.832		0.315	0.920	0.054–15.757	0.954	0.687	.0100–4.719	0.703
Both	6.061	1.275–28.816		0.024	5.520	0.617–49.393	0.127	2.500	0.191–32.802	0.485
Balloon dilatation	3.975	1.416–11.156		0.009	9.789	1.093–87.705	0.041	18.857	1.926–184.631	0.012
Adverse events of stents	0.729	0.209–2.537		0.619	0.591	0.132–2.642	0.491	0.960	0.090–10.224	0.973
With bile leakage	1.051	0.092–12.051		0.968	-	-	1.000	0.214	0.012–3.928	0.299
With stones	1.598	0.590–4.327		0.357	1.750	0.417–7.346	0.445	3.500	0.637–19.238	0.150
Adverse events of ERCP	1.495	0.549–4.074		0.432	1.543	0.499–4.771	0.452	-	-	0.999

^a^Univariate analysis was used

NAS, non-anastomotic strictures; AS, anastomotic strictures; BMI, body mass index; LT, liver transplantation; DBD, donation after brain death; GDA, gastroduodenal artery; ERCP, endoscopic retrograde cholangiopancreatography; MPS, multiple plastic stents; FCSEMS, fully covered self-expanding metal stent

### Survival analysis

**Fig. 3:**
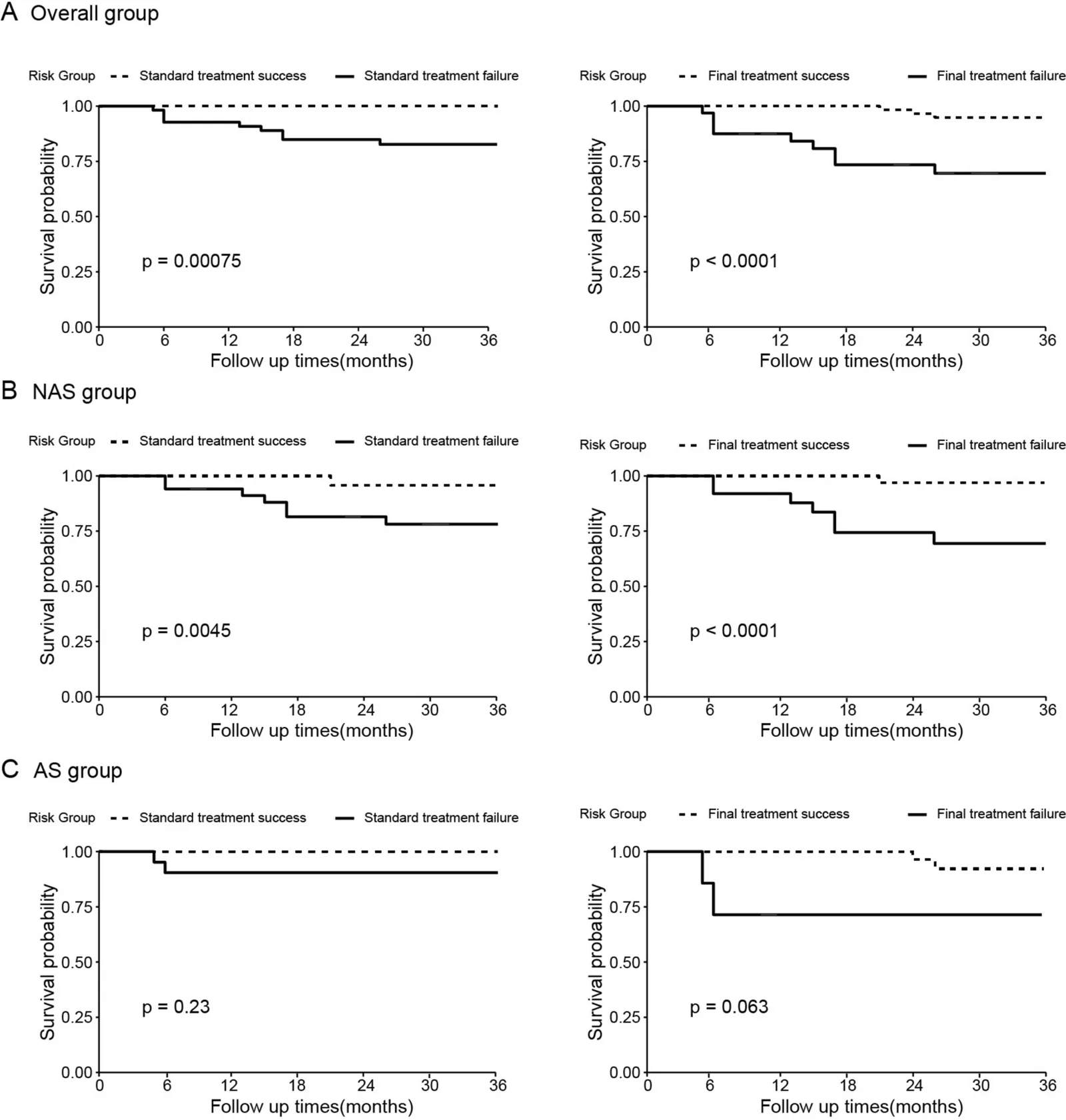
Survival according to achievement of standard treatment success and treatment success in overall group (**A**), NAS group (**B**) and AS group (**C**)

After 51.2 months of follow-up after diagnosis of biliary strictures, the overall survival was 86% (80/93), with the survival of 80.7% (46/57) in the NAS group and 94.6% (35/37) in the AS group. Kaplan–Meier analysis showed that in the overall group and the NAS group, patients who achieved standard treatment success and final treatment success had a better overall survival benefit (*P* < 0.05, Fig. 3). In the AS group, although the Kaplan–Meier analysis showed that the P value did not reach statistical significance, the survival curve showed a clear trend towards separation. Therefore, compared to patients who did not achieve standard treatment success and final treatment success, patients with two treatment success patterns have a better long-term overall survival.

## Discussion

This study evaluated the effectiveness and impact of ERCP in the treatment of NAS and AS following LT. The results showed no statistically significant difference in the treatment success rate between NAS and AS within the standard ERCP treatment protocol. However, when looking at final treatment success, AS showed a significantly higher success rate compared to NAS. Notably, the combination of stent placement with balloon dilatation during the ERCP procedure led to a significant increase in the final treatment success rate for both NAS and AS patients. In addition, it was interesting to note that for patients with either NAS or AS, achieving both standard and final treatment success was associated with prolonged survival. These findings underscore the importance of ERCP in the management of post-LT strictures and highlight the potential benefits of incorporating stenting with balloon dilation to improve outcomes.

The standardized treatment success rate for NAS was 40.4%, and the final treatment success rate was 56.1%, while in the AS group, the incidences of the two treatment success patterns were 41.7% and 80.6%. This study found that although there was no significant difference in the treatment success rate between NAS and AS in the standard ERCP treatment protocol, the success rate of AS was significantly higher than that of NAS in terms of the final treatment outcome, which measured overall efficacy. This result was consistent with previous research findings, indicating that NAS was more difficult to treat and had a lower success rate [11, 20, 21]. Analyzing the reasons, the pathological mechanism of NAS is complex, involving extensive inflammation, fibrosis, or autoimmune reactions, and its location and morphology are varied, which increases the difficulty of treatment. In contrast, the pathological changes of AS are relatively simple, and the treatment is more targeted [12, 22]. In addition, the high recurrence rate after NAS treatment, multiple underlying diseases in patients, and the lack of a unified treatment standard are also important factors leading to a low success rate of treatment [23].

The European Society of Gastrointestinal Endoscopy (ESGE) advocates for stent implantation or balloon dilation combined with stent implantation, as the primary therapeutic option for biliary stricture following LT [13]. This recommendation aligns with the standard ERCP protocol adopted at our institution, given that balloon dilation alone is associated with elevated recurrence rates and a heightened risk of biliary rupture [4]. While stent implantation may elevate the occurrence of postoperative complications, it notably diminishes the rates of stenosis persistence and recurrence [24]. Costamagna et al. [25] reported a success rate of 89% for the combined treatment regimen of stent implantation and balloon dilation. Furthermore, Keane et al. [7] underscored a prevalent tendency among studies to favor the combined use of balloon dilation and stent implantation as the frontline strategy for addressing such stricture. Our study findings corroborated that, in comparison to standalone stent implantation, the combination therapy of stent implantation and balloon dilation boasted a superior final treatment success rate, mirroring the conclusions drawn from prior research endeavors.

Besides, the selection of stents poses another contemporary challenge. Prior research has indicated MPS as the preferred option, with FCSEMS being reserved primarily for the routine management of refractory stricture in AS [20, 26]. Conversely, the American Society for Gastrointestinal Endoscopy (ASGE) advocates FCSEMS as the preferred endoscopic approach for treating extrahepatic bile duct stricture [9]. Kaffes et al. [27] reported that there was no statistically significant difference in stenosis regression and recurrence rates between FCSEMS and MPS. Furthermore, Martins et al. [16] discovered that the average treatment cost associated with FCSEMS was lower compared to MPS. Our study revealed that the combined use of FCSEMS and MPS enhanced the final treatment success rate for all patients. It is worth noting that we did not stratify patients based on stent type, as the decision for stent placement during ERCP treatment was contingent upon the endoscopists’ clinical judgment and experience. Therefore, further research is essential to corroborate these findings.

Our research further uncovered that both achieving standard treatment success and final treatment success significantly enhanced the survival expectancy of NAS patients. Indeed, successful NAS treatment post-transplantation not only elevates the quality of life for these patients but also effectively wards off graft failure, thereby mitigating the risks of re-transplantation or mortality [28, 29]. However, in the case of AS, despite the Kaplan–Meier analysis indicating that the P-value failed to attain statistical significance, the survival curve exhibited a distinct divergence trend. Consequently, we hypothesize that AS patients who attain both standard treatment success and final treatment success are likely to exhibit superior long-term overall survival rates compared to those who fall short of these benchmarks.

Our study has several limitations that merit attention. First, as a retrospective study, it lacked randomization of patients undergoing ERCP treatment, which may have introduced selection bias. Secondly, patient diagnosis was made on the basis of MRCP results prior to the final ERCP procedure, with the possibility of patients transitioning from AS to NAS, which compromises the robustness of our results. Thirdly, the selection of ERCP treatment procedures was based on the experience of the endoscopist, without a standardized treatment protocol, which may have influenced the study results. Finally, the sample size was relatively small, which may limit the generalizability of our findings.

## Conclusion

In conclusion, ERCP offers a relatively safe and effective therapeutic option for NAS and AS patients, significantly prolonging their long-term survival. Besides, balloon dilatation combined with stent implantation can promote ERCP treatment success. Future endeavors should also prioritize assessing the quality of life of patients post-ERCP, while actively exploring innovative technologies and methodologies to refine the treatment process and maximize patient benefits. We aim to establish standardized treatment protocols and conduct larger prospective studies to further validate our findings and improve patient outcomes. Through such sustained efforts, we anticipate providing NAS and AS patients with even more personalized, efficient, and secure medical interventions.

## Supplementary Information

Below is the link to the electronic supplementary material.

Supplementary file1 (DOCX 2672 KB)

## Data Availability

Data is available from the corresponding author upon reasonable request. The data are not publicly available since this could compromise the privacy of research participants.

## References

[1] Navez J, Iesari S, Kourta D et al (2021) The real incidence of biliary tract complications after adult liver transplantation: the role of the prospective routine use of cholangiography during post-transplant follow-up. Transpl Int 34:245–258. 10.1111/tri.13786 33188645

[2] Seehofer D, Eurich D, Veltzke-Schlieker W et al (2013) Biliary complications after liver transplantation: old problems and new challenges. Am J Transplant 13:253–265. 10.1111/ajt.12034 23331505

[3] Shin M, Joh JW (2016) Advances in endoscopic management of biliary complications after living donor liver transplantation: comprehensive review of the literature. World J Gastroenterol 22:6173–6191. 10.3748/wjg.v22.i27.6173 PMC4945977 27468208

[4] Magro B, Tacelli M, Mazzola A et al (2021) Biliary complications after liver transplantation: current perspectives and future strategies. Hepatobiliary Surg Nutr 10:76–92. 10.21037/hbsn.2019.09.01 PMC7867735 33575291

[5] den op Dries S, Westerkamp AC, Karimian N et al (2014) Injury to peribiliary glands and vascular plexus before liver transplantation predicts formation of non-anastomotic biliary strictures. J Hepatol 60:1172–1179. 10.1016/j.jhep.2014.02.010 24560661

[6] Bofill A, Cárdenas A (2024) A practical approach to the endoscopic management of biliary strictures after liver transplantation. Ann Hepatol 29:101186. 10.1016/j.aohep.2023.101186 38035999

[7] Keane MG, Devlin J, Harrison P et al (2021) Diagnosis and management of benign biliary strictures post liver transplantation in adults. Transplant Rev 35:100593. 10.1016/j.trre.2020.100593 33388638

[8] Larghi A, Tringali A, Rimbaş M et al (2019) Endoscopic management of benign biliary strictures after liver transplantation. Liver Transpl 25:323–335. 10.1002/lt.25358 30329213

[9] Kohli DR, Amateau SK, Desai M et al (2023) American Society for Gastrointestinal Endoscopy guideline on management of post-liver transplant biliary strictures: summary and recommendations. Gastrointest Endosc 97:607–614. 10.1016/j.gie.2022.10.007 36797162

[10] Heinemann M, Tafrishi B, Pischke S et al (2019) Endoscopic retrograde cholangiography and percutaneous transhepatic cholangiodrainage in biliary strictures after liver transplantation: long-term outcome predictors and influence on patient survival. Liver Int 39:1155–1164. 10.1111/liv.13995 30367552

[11] Zhang CC, Rupp C, Exarchos X et al (2023) Scheduled endoscopic treatment of biliary anastomotic and nonanastomotic strictures after orthotopic liver transplantation. Gastrointest Endosc 97:42–49. 10.1016/j.gie.2022.08.034 36041507

[12] Wu Z, Liu D, Ou Y et al (2024) Mechanism and endoscopic-treatment-induced evolution of biliary non-anastomotic stricture after liver transplantation revealed by single-cell RNA sequencing. Clin Transl Med 14:e1622. 10.1002/ctm2.1622 PMC10938070 38481381

[13] Dumonceau JM, Tringali A, Papanikolaou IS et al (2018) Endoscopic biliary stenting: indications, choice of stents, and results: European Society of Gastrointestinal Endoscopy (ESGE) clinical guideline - updated October 2017. Endoscopy 50:910–930. 10.1055/a-0659-9864 30086596

[14] Albert JG, Filmann N, Elsner J et al (2013) Long-term follow-up of endoscopic therapy for stenosis of the biliobiliary anastomosis associated with orthotopic liver transplantation. Liver Transpl 19:586–593. 10.1002/lt.23643 23585381

[15] Tal AO, Finkelmeier F, Filmann N et al (2017) Multiple plastic stents versus covered metal stent for treatment of anastomotic biliary strictures after liver transplantation: a prospective, randomized, multicenter trial. Gastrointest Endosc 86:1038–1045. 10.1016/j.gie.2017.03.009 28302527

[16] Martins FP, De Paulo GA, Contini MLC et al (2018) Metal versus plastic stents for anastomotic biliary strictures after liver transplantation: a randomized controlled trial. Gastrointest Endosc 87:131.e1-131.e13. 10.1016/j.gie.2017.04.013 28455159

[17] Tringali A, Tarantino I, Barresi L et al (2019) Multiple plastic versus fully covered metal stents for managing post-liver transplantation anastomotic biliary strictures: a meta-analysis of randomized controlled trials. Ann Gastroenterol 32:407–415. 10.20524/aog.2019.0376 PMC6595920 31263364

[18] Buxbaum JL, Freeman M, Amateau SK et al (2023) American Society for Gastrointestinal Endoscopy guideline on post-ERCP pancreatitis prevention strategies: methodology and review of evidence. Gastrointest Endosc 97:163-183.e40. 10.1016/j.gie.2022.09.011 36517309

[19] Elmunzer BJ, Maranki JL, Gómez V et al (2023) ACG clinical guideline: diagnosis and management of biliary strictures. Am J Gastroenterol 118:405–426. 10.14309/ajg.0000000000002190 36863037

[20] Roos FJM, Poley JW, Polak WG et al (2017) Biliary complications after liver transplantation; recent developments in etiology, diagnosis and endoscopic treatment. Best Pract Res Clin Gastroenterol 31:227–235. 10.1016/j.bpg.2017.04.002 28624111

[21] Nemes B, Gámán G, Doros A (2015) Biliary complications after liver transplantation. Expert Rev Gastroenterol Hepatol 9:447–466. 10.1586/17474124.2015.967761 25331256

[22] Jung DH, Ikegami T, Balci D et al (2020) Biliary reconstruction and complications in living donor liver transplantation. Int J Surg. 10.1016/j.ijsu.2020.04.069 32387205

[23] van Rijn R, Schurink IJ, de Vries Y et al (2021) Hypothermic machine perfusion in liver transplantation - a randomized trial. N Engl J Med 384:1391–1401. 10.1056/NEJMoa2031532 33626248

[24] Kao D, Zepeda-Gomez S, Tandon P et al (2013) Managing the post-liver transplantation anastomotic biliary stricture: multiple plastic versus metal stents: a systematic review. Gastrointest Endosc 77:679–691. 10.1016/j.gie.2013.01.015 23473000

[25] Costamagna G, Pandolfi M, Mutignani M et al (2001) Long-term results of endoscopic management of postoperative bile duct strictures with increasing numbers of stents. Gastrointest Endosc 54:162–168. 10.1067/mge.2001.116876 11474384

[26] Lee HW, Shah NH, Lee SK (2017) An update on endoscopic management of post-Liver transplant biliary complications. Clin Endosc 50:451–463. 10.5946/ce.2016.139 PMC5642064 28415168

[27] Kaffes A, Griffin S, Vaughan R et al (2014) A randomized trial of a fully covered self-expandable metallic stent versus plastic stents in anastomotic biliary strictures after liver transplantation. Ther Adv Gastroenterol 7:64–71. 10.1177/1756283x13503614 PMC3903084 24587819

[28] Guo Z, Zhao Q, Jia Z et al (2023) A randomized-controlled trial of ischemia-free liver transplantation for end-stage liver disease. J Hepatol 79:394–402. 10.1016/j.jhep.2023.04.010 37086919

[29] Pan B, Liu W, Ou YJ et al (2023) Gastroduodenal artery disconnection during liver transplantation decreases non-anastomotic stricture incidence. Hepatobiliary Pancreat Dis Int 22:28–33. 10.1016/j.hbpd.2022.09.013 36210313

