# Supplementary material for: Endoscopic treatment of biliary strictures after liver transplantation: balloon dilatation with stenting improves long-term outcomes
Source: Updates Surg. 2026 Feb 6;78(6):2587–96. doi: 10.1007/s13304-026-02534-9 (PMC13736480; doi:10.1007/s13304-026-02534-9)
Supplement: Supplementary file 1 [file 13304_2026_2534_MOESM1_ESM.docx]

Table S1. Laboratory parameters at different treatment stage

| Characteristics | Overall groups  N = 93 | NAS groups  N = 57 | AS groups  N = 36 |
| --- | --- | --- | --- |
| Pre-transplantation | | | |
| WBC (10^9^/L) | 5.6 ± 3.5 | 5.7 ± 3.6 | 5.5 ± 3.4 |
| Hb (g/L) | 107.1 ± 28.9 | 105.5 ± 29.5 | 109.8 ± 28.1 |
| PLT (10^9^/L) | 88.8 ± 57.1 | 92.4 ± 61.1 | 83.0 ± 50.4 |
| ALT (IU/L) | 104.7 ± 152.9 | 97.8 ± 150.7 | 115.6 ± 157.8 |
| AST (IU/L) | 107.5 ± 139.6 | 96.8 ± 126.8 | 124.5 ± 158.3 |
| ALB (g/L) | 36.5 ± 7.8 | 36.1 ± 8.5 | 37.1 ± 6.8 |
| TBIL (μmol/L) | 168.9 ± 179.1 | 161.3 ± 171.5 | 181.0 ± 192.5 |
| Cr (μmol/L) | 76.6 ± 49.2 | 76.5 ± 45.5 | 76.8 ± 55.3 |
| PT (seconds) | 16.8 ± 5.8 | 16.5 ± 5.2 | 17.5 ± 6.7 |
| Pre- ERCP | | | |
| WBC (10^9^/L) | 5.4 ± 4.7 | 5.6 ± 5.3 | 5.1 ± 3.7 |
| Hb (g/L) | 111.0 ± 24.7 | 108.2 ± 24.8 | 115.4 ± 24.2 |
| PLT (10^9^/L) | 162.8 ± 87.2 | 160.2 ± 81.7 | 166.9 ± 96.6 |
| ALT (IU/L) | 116.7 ± 110.3 | 118.5 ± 113.0 | 114.0 ±107.5 |
| AST (IU/L) | 93.4 ± 77.4 | 92.7 ± 73.5 | 94.4 ± 84.3 |
| ALB (g/L) | 38,8 ± 5.3 | 38.1 ± 5.0 | 39.8 ± 5.8 |
| TBIL (μmol/L) | 83.5 ± 94.2 | 84.0 ± 81.0 | 82.7 ± 113.3 |
| Cr (μmol/L) | 80.5 ± 33.6 | 77.1 ± 30.6 | 85.9 ± 37.7 |
| PT (seconds) | 11.9 ± 4.7 | 12.1 ± 5.9 | 11.4 ± 1.4 |
| During ERCP | | | |
| WBC (10^9^/L) | 5.1 ± 2.8 | 5.3 ± 3.1 | 4.7 ± 2.3 |
| Hb (g/L) | 119.1 ± 25.1 | 115.6 ± 25.6 | 124.6 ± 23.7 |
| PLT (10^9^/L) | 167.8 ± 89.3 | 164.0 ± 92.4 | 173.9 ± 85.1 |
| ALT (IU/L) | 54.8 ± 64.5 | 57.4 ± 67.8 | 50.8 ± 59.6 |
| AST (IU/L) | 54.4 ± 48.1 | 61.2 ± 50.4 | 43.7 ± 42.6 |
| ALB (g/L) | 39.5 ± 5.9 | 38.3 ± 6.4 | 41.4 ± 4.6 |
| TBIL (μmol/L) | 62.0 ± 107.2 | 81.7 ± 131.5 | 30.8 ± 305. |
| Cr (μmol/L) | 91.0 ± 72.9 | 91.9 ± 87.8 | 89.7 ± 40.7 |
| PT (seconds) | 12.1 ± 72.9 | 12.5 ± 6.3 | 11.4 ± 1.4 |
| End-follow-up |  |  |  |
| WBC (10^9^/L) | 6.0 ± 4.4 | 6.2 ± 5.3 | 5.7 ± 2.2 |
| Hb (g/L) | 124.0 ± 26.8 | 118.5 ± 28.6 | 132.8 ± 21.3 |
| PLT (10^9^/L) | 156.5 ± 71.2 | 153.3 ± 78.5 | 161.7 ± 59.5 |
| ALT (IU/L) | 40.4 ± 51.1 | 49.0 ± 63.6 | 26.8 ± 18.7 |
| AST (IU/L) | 46.9 ± 46.4 | 55.5 ± 54.7 | 33.4 ± 23.6 |
| ALB (g/L) | 39.2 ± 6.0 | 37.8 ± 6.4 | 41.5 ± 4.6 |
| TBIL (μmol/L) | 54.7 ± 100.4 | 75.3 ± 123.4 | 22.1 ± 18.4 |
| Cr (μmol/L) | 96.9 ± 81.3 | 103.2 ± 99.6 | 87.0 ± 36.6 |
| PT (seconds) | 12.0 ± 2.5 | 12.3 ± 3.0 | 11.4 ± 1.4 |

ERCP, [endoscopic retrograde cholangiopancreatography](https://www.sciencedirect.com/topics/medicine-and-dentistry/endoscopic-retrograde-cholangiopancreatography); WBC, white blood cell; Hb, hemoglobin; PLT, platelet; ALT, alanine aminotransferase; AST, aspartate aminotransferase; ALB, albumin; Cr, creatinine; PT, prothrombin time; TBIL, total bilirubin

Fig. S1. Course of liver function indicators over time compared for patients with and without achievement of standard treatment success (A) and final treatment success (B) in overall group

Fig. S2. Course of liver function indicators over time compared for patients with and without achievement of standard treatment success (A) and final treatment success (B) in NAS group

Fig. S3. Course of liver function indicators over time compared for patients with and without achievement of standard treatment success (A) and final treatment success (B) in AS group
